# Development of an electronic medical record-based algorithm to identify patients with Stevens-Johnson syndrome and toxic epidermal necrolysis in Japan

**DOI:** 10.1371/journal.pone.0221130

**Published:** 2019-08-13

**Authors:** Toshiki Fukasawa, Hayato Takahashi, Norin Kameyama, Risa Fukuda, Shihori Furuhata, Nanae Tanemura, Masayuki Amagai, Hisashi Urushihara

**Affiliations:** 1 Division of Drug Development and Regulatory Science, Graduate School of Pharmaceutical Sciences, Keio University, Shibakoen, Minato-ku, Tokyo, Japan; 2 Department of Dermatology, Keio University School of Medicine, Shinanomachi, Shinjuku-ku, Tokyo, Japan; Northwestern University Feinberg School of Medicine Galter Health Sciences Library, UNITED STATES

## Abstract

Stevens-Johnson syndrome (SJS) and toxic epidermal necrolysis (TEN), severe drug reactions, are often misdiagnosed due to their rarity and lack of information on differential diagnosis. The objective of the study was to develop an electronic medical record (EMR)-based algorithm to identify patients with SJS/TEN for future application in database studies. From the EMRs of a university hospital, two dermatologists identified all 13 patients with SJS/TEN seen at the Department of Dermatology as the case group. Another 1472 patients who visited the Department of Dermatology were identified using the ICD-10 codes for diseases requiring differentiation from SJS/TEN. One hundred of these patients were then randomly sampled as controls. Based on clinical guidelines for SJS/TEN and the experience of the dermatologists, we tested 128 algorithms based on the use of ICD-10 codes, clinical courses for SJS/TEN, medical encounters for mucocutaneous lesions from SJS/TEN, and items to exclude paraneoplastic pemphigus. The sensitivity, specificity, positive predictive value (PPV), negative predictive value (NPV), and diagnostic odds ratio (DOR) of each algorithm were calculated, and the optimal algorithm was defined as that with high PPV and maximal sensitivity and specificity. One algorithm, consisting of a combination of clinical course for SJS/TEN, medical encounters for mucocutaneous lesions from SJS/TEN, and items to exclude paraneoplastic pemphigus, but not ICD-10 codes, showed a sensitivity of 76.9%, specificity of 99.0%, PPV of 40.5%, NPV of 99.8%, and DOR of 330.00. We developed a potentially optimized algorithm for identifying SJS/TEN based on clinical practice records. The almost perfect specificity of this algorithm will prevent bias in estimating relative risks of SJS/TEN in database studies. Considering the small sample size, this algorithm should be further tested in different settings.

## Introduction

Stevens-Johnson syndrome (SJS) and toxic epidermal necrolysis (TEN) are rare life-threatening diseases characterized by epidermal detachment and mucosal lesions, and are mainly induced by medications [1]. SJS and TEN are considered one disease entity, and differ in the percentage of body surface area affected: in Japan, SJS is defined as <10% affected and TEN as ≥10% [2]. Estimated incidence in Japan is 3.1 for SJS and 1.3 for TEN per million per year, and epidemiological evidence for individual suspect drugs remains limited [3]. Carbamazepine, an anticonvulsant, is known to be associated with the risk of SJS/TEN, and polymorphism in human leukocyte antigen (HLA) type is known to be associated with sensitivity to carbamazepine-induced SJS/TEN. The distribution of this phenotype differs between Japanese and the other ethnic groups [4–6]. The risk of SJS/TEN in Japanese populations should therefore be investigated separately.

The recent availability in Japan of several large-scale healthcare databases, primarily administrative claims databases or electronic medical records (EMRs), for postmarketing observational studies has potentially enabled the detection of rare and serious adverse drug reactions, whose estimates of absolute and relative risk in a defined population, i.e. patient group treated with a particular drug, have not been available [7]. Observational studies for SJS/TEN using such databases may be susceptible to biases by misdiagnosis, a frequent occurrence with these conditions due to their rarity, lack of information on differential diagnosis, and use of tentative disease codes intended to allow reimbursement claims for diagnostic testing. Several previous studies have developed the criteria or algorithms to identify SJS/TEN from healthcare databases of the US and UK [8–11]. However, it is difficult to apply their methods to epidemiological studies using medical information databases in Japan because of the differences in healthcare systems between Japan and other countries. In addition, linkage of routinely collected health data (RCD) sets such as insurance claims and administrative databases to patients’ medical charts and disease registry data at the individual level requires an opt-in process for secondary use, making validation of electronic codes used for these large-scale databases almost impossible in Japan. A single-center study to develop an algorithm applicable to RCD is accordingly one of the few feasible options. To date, many studies have attempted to develop algorithms to identify health outcomes from databases at single centers throughout the world [12–21].

Here, to assess the quantitative risk of SJS/TEN from drugs of concern, we developed algorithms to identify SJS/TEN patients using the EMRs of a university hospital in Japan.

## Methods

### Study design and setting

The study was conducted under a retrospective cross-sectional design in SJS/TEN case subjects and controls with diseases requiring differential diagnosis from SJS/TEN. Subjects were selected from among patients seen at the Department of Dermatology, Keio University Hospital, between January 1, 2012, and August 7, 2017. Keio University Hospital is a 1044-bed tertiary medical care, university-affiliated, educational hospital located in central Tokyo. It served 816242 outpatients, 291603 inpatients and 16239 emergency patients in 2016.

### Study population

Two experienced, board-certified dermatologists (HT and RF) identified all 14 patients with SJS/TEN seen and diagnosed at the Department of Dermatology by reviewing medical records in the patients’ EMRs. After excluding these 14 true SJS/TEN cases, the controls were sampled from the other 2149 patients who had ICD-10 codes for SJS/TEN or the following diseases, which were considered to be diseases requiring differential diagnosis from SJS/TEN, at the Department of Dermatology (S1 Table): toxic shock syndrome (TSS), staphylococcal scalded skin syndrome (SSSS), impetigo contagiosa, acute generalized exanthematous pustulosis (AGEP), paraneoplastic pemphigus (PNP), drug eruption, drug-induced hypersensitivity syndrome (DIHS), toxicoderma, bullous erythema multiforme, and erythema multiforme major (EMM). The above diseases were determined to be distinguished from SJS/TEN by reference to the Japanese clinical guidelines and expert dermatologists’ opinion [2]. After excluding 677 patients who had been given these diagnoses for testing purposes only, we randomly selected 100 control patients from the remaining 1472 for EMR review.

The study was approved by the ethics committees of Keio University Faculty of Pharmacy and Keio University School of Medicine (registration number: 180831–6 and 20170133, respectively) and conducted according to the local ethical guidance for medical research involving human subjects. Because of the retrospective nature of the study, the requirement for written informed consent was waived and an opportunity was offered to opt-out through the website of Keio University Hospital.

### Algorithm development

Development of the algorithms was based on clinical guidelines for SJS/TEN issued by a research committee for intractable diseases officially appointed by the Ministry of Health, Labour and Welfare of Japan and the expertise of two experienced dermatologists, one of whom is a committee member (HT) [2]. We developed four EMR-based algorithm sets, A to D, with four domains: Domain 1 included diagnosis of SJS/TEN at the Department of Dermatology; Domain 2 included items related to the clinical course of SJS/TEN; Domain 3 included items related to medical encounters for mucocutaneous lesions from SJS/TEN; and Domain 4 included items to exclude the differential diagnosis of PNP (Table 1). Each set encompassed 32 patterns of combination of the algorithm items: sets A and C used Domain 1 but B and D did not; and sets A and B used item 6a of Domain 4 but C and D used 6b. When an applicable item was found during a hospital visit or hospitalization period which included SJS/TEN or the differential diagnoses, the item was considered “yes”. All items other than Item 1 were dichotomous variables, giving 32 combinations of Items 2 to 6. Details of these and the 128 algorithms developed using them are shown in Table 1 and S3–S6 Tables, respectively. 128 algorithms were applied to case and control patients, and a performance index was calculated for each. The optimal algorithm was determined based on a high positive predictive value (PPV) while maximizing sensitivity and specificity. We also tested the performance of another 1086 algorithms (algorithm set E), which included all possible combinations using any of items 1–6b, excluding those for sets A–D (S7 and S8 Tables).

**Table 1:** Details of algorithms used to identify patients with Stevens-Johnson syndrome and toxic epidermal necrolysis.

Algorithm domain and item (date required for each item)	Algorithm definition			
	Set A	Set B	Set C	Set D
**Domain 1) Diagnosis of SJS/TEN at the Department of Dermatology**				
Item 1. Records show ICD-10 code for SJS/TEN (L51.1 or L51.2) at the Department of Dermatology (admission and discharge date required)	yes	N/A	yes	N/A
**Domain 2) Clinical course for SJS/TEN**				
Item 2: Hospitalization^a^ (admission and discharge date required)	yes/no	yes/no	yes/no	yes/no
Item 3: Skin biopsy^b^ (date of biopsy required)	yes/no	yes/no	yes/no	yes/no
Item 4: Systemic treatments for SJS/TEN^c^ (order date required) Receiving any one of the following treatments: steroid therapy (≥0.5 mg/kg/day of prednisolone), intravenous immunoglobulin therapy, or plasma exchange therapy	yes/no	yes/no	yes/no	yes/no
**Domain 3) Medical encounters for mucocutaneous lesions from SJS/TEN**				
Item 5: Medical encounters included any of the following^d^ (if any item is answered “yes”, Item 5 is considered as “yes”)	yes/no	yes/no	yes/no	yes/no
• Records of ICD-10 code for SJS/TEN at the Department of Ophthalmology or Dentistry, or for mucocutaneous lesions^e^ (no limitation on department) (date of diagnosis with ICD-10 code required)				
• Steroid eyedrops or ophthalmic ointment^f^ (order date required)				
• Slit-lamp microscopy (date of microscopy required)				
**Domain 4) Exclusion of paraneoplastic pemphigus (either 6a or 6b)**				
Item 6a: Prescription of anti-cancer drug^g^ (order date required)	yes/no	yes/no	N/A	N/A
Item 6b: Anti-desmoglein 1 or 3 antibody test ≥2^h^ (execution date required)	N/A	N/A	yes/no	yes/no

SJS, Stevens-Johnson syndrome; TEN, toxic epidermal necrolysis; ICD-10, International Classification of Diseases, 10th Edition.

^a^ Inpatient treatment of SJS/TEN is recommended [2].

^b^ Establishing a diagnosis of SJS/TEN requires skin biopsy to determine massive epidermal degeneration [22].

^c^ Steroid therapy (≥0.5 mg/kg/day of prednisolone), intravenous immunoglobulin therapy (IVIg), or plasma exchange therapy are recommended for SJS/TEN patients in Japan [23–25].

^d^ Item 5 consists of the following three components relevant to medical encounters for mucocutaneous lesions, given that SJS/TEN often cause mucous membrane lesions, including ocular, oral and genital symptoms [1].

^e^ See S2 Table.

^f^ Steroid eyedrops or ophthalmic ointment are used for acute eye lesions [26, 27].

^g^ Since PNP is a complication of neoplasm, anti-cancer drugs are usually used for the neoplastic condition.

^h^ Differential diagnosis for PNP generally requires multiple testing of two times or more [28].

### Medical chart review

Two trained reviewers with pharmaceutical expertise conducted a manual EMR review at Keio University Hospital. The following information was collected: patient characteristics (sex, date of birth), discharge summary (admission and discharge date), diagnosis [final diagnosis made by a physician, and diagnose code according to the International Classification of Diseases, 10th Edition (ICD-10)], medication orders (order date, medication name, and daily dose), and procedure orders (order date, procedure name, and department in charge of the procedure). Of the 14 case-patients, one with TEN who was referred from another hospital where acute treatment had been completed was excluded. For outpatients, we collected information on Items 1 and 3–5 used in the algorithms (Table 1) on the date the disease diagnosis codes shown in S1 Table were first recorded (i.e., index date). For inpatients, we collected information on Item 2 as well as those above between one day before the index date and the discharge date. We collected information on Item 6a for 30 days before the first day of treatment in the Department of Dermatology, regardless of the presence of hospital admission. For patients with anti-desmoglein 1 (Dsg1) or Dsg3 antibody testing (Item 6b) at least once during the observation period, we checked whether they had undergone the test multiple times in the 180 days after the first test regardless of the presence of hospital admission, because the test reimbursement claims are issued only once a month.

### Statistical analysis

Subject characteristics were summarized using descriptive statistics by group. Performance indices, including sensitivity, specificity, PPV, negative predictive value (NPV), and diagnostic odds ratio (DOR) as well as their 95% confidence intervals (CIs) were computed for each algorithm [29]. We identified all SJS/TEN patients diagnosed and treated at the Department of Dermatology; calculation of sensitivity was therefore straightforward, as follows:

$$\mathrm{Sensitivity}=\frac{\mathrm{True}\;\mathrm{Positive}\;[\mathrm{TP}]}{\mathrm{True}\;\mathrm{Positive}\;[\mathrm{TP}]+\mathrm{False}\;\mathrm{Negative}\;[\mathrm{FN}]}$$

Given that sampling of control patients, specificity, PPV, NPV, and DOR were calculated by weighting the number of FPs and TNs with the sampling proportion (i.e., 100/1472). Specificity, PPV, and NPV were calculated as follows:

$$\mathrm{Specificity}=\frac{\mathrm{True}\;\mathrm{Negative}\;[\mathrm{TN}]\times 1472/100}{(\mathrm{True}\;\mathrm{Negative}\;[\mathrm{TN}]+\mathrm{False}\;\mathrm{Positive}\;[\mathrm{FP}])\times 1472/100}$$

$$\mathrm{PPV}=\frac{\mathrm{TP}}{\mathrm{TP}+\mathrm{FP}\times 1472/100}$$

$$\mathrm{NPV}=\frac{\mathrm{TN}\times 1472/100}{\mathrm{TN}\times 1472/100+\mathrm{FN}}$$

DOR is the ratio of the odds of having a positive algorithm result in case patients relative to the odds of a positive algorithm result in control patients [29]. DOR was calculated as follows:

$$\mathrm{DOR}=\frac{\mathrm{TP}/\mathrm{FN}}{(\mathrm{FP}\times 1472/100)/(\mathrm{TN}\times 1472/100)}$$

A high DOR indicates a greater likelihood of being positive in cases than being positive in controls.

The 95% CIs of the proportions were calculated using the Wilson score method [30]. The 95% CIs of the DORs were calculated using Woolf’s method with Haldane’s correction [31]. All statistical analyses were performed with SAS version 9.4 (SAS Institute Inc., Cary, NC, USA).

## Results

### Characteristics of the study population

The 13 case and 100 control patients had a mean (SD) age of 56.4 (15.9) and 52.9 (21.8) years, respectively (Table 2). There were eight men (61.5%) in the case and 41 (41.0%) in the control group. The distribution of ICD-10 codes in the case and control patients is shown in S9 and S10 Tables. The control patients with TSS, SSSS, AGEP, PNP, or nonbullous erythema multiforme were not sampled because there were no or only a few patients in the source population. Eight cases (61.5%) had ICD-10 codes for SJS/TEN and 85 controls (85.0%) had accurate ICD-10 codes, consistent with the diagnosis by the attending physician at the Department of Dermatology. All cases were hospitalized (100%, Item 2), 12 had skin biopsy (92.3%, Item 3), 12 received systemic treatment for SJS/TEN (92.3%, Item 4), and 12 had medical encounters for mucocutaneous lesions from SJS/TEN (92.3%, Item 5). Thirty-two controls were hospitalized (32.0%, Item 2), 12 had skin biopsy (12.0%, Item 3) and 12 received anti-cancer drug treatment (12.0%, Item 6a). The algorithm items and their chronological order in the 13 SJS/TEN cases are shown in S11 Table.

**Table 2:** Characteristics of the study population.

	Case group		Control group	
Number of patients	13	(100)	100	(100)
Sex				
Male	8	(61.5)	41	(41.0)
Female	5	(38.5)	59	(59.0)
Age, years	56.4	(15.9)	52.9	(21.8)
Agreement between diagnosis with ICD-10 code and final diagnosis	8	(61.5)	85	(85.0)
Referral from another hospital	1	(7.7)	24	(24.0)
Clinical course for SJS/TEN at the Department of Dermatology				
Item 2: Hospitalization	13	(100)	32	(32.0)
Item 3: Skin biopsy	12	(92.3)	12	(12.0)
Item 4: Systemic treatment for SJS/TEN	12	(92.3)	8	(8.0)
Steroid therapy	12	(92.3)	8	(8.0)
Intravenous immunoglobulin therapy	3	(23.1)	0	(0.0)
Plasma exchange therapy	0	(0.0)	0	(0.0)
Medical encounters for mucocutaneous lesions from SJS/TEN				
Item 5: Presence of medical encounters for mucocutaneous lesions from SJS/TEN	12	(92.3)	6	(6.0)
ICD-10 code for SJS/TEN at the Department of Ophthalmology or Dentistry, or for mucocutaneous lesions (no limitation on department)	11	(84.6)	2	(2.0)
Steroid eyedrops or ophthalmic ointment	8	(61.5)	2	(2.0)
Slit-lamp microscopy	10	(76.9)	6	(6.0)
Exclusion of paraneoplastic pemphigus				
Item 6a: Prescription of anti-cancer drug	2	(15.4)	12	(12.0)
Item 6b: Anti-desmoglein 1 or 3 antibody test ≥2	0	(0.0)	2	(2.0)

ICD-10, International Classification of Diseases, 10th Edition; SJS, Stevens-Johnson syndrome; TEN, toxic epidermal necrolysis.

Data are presented as mean (SD) or number of subjects (%).

### Performance of algorithms

Of 128 algorithms, 37 were applicable to at least one case or control patient (Table 3). The others detected neither cases nor controls (S12 Table). Algorithms A09, B01, and B09 had the highest DOR (353.40; 95% CI, 13.72–9102.13) with a sensitivity of 7.7% (95% CI: 1.4–33.3%), specificity of 100.0% (95% CI: 99.7–100.0%), PPV of 100.0% (95% CI: 20.7–100.0%) and NPV of 99.2% (95% CI: 98.6–99.5%). Algorithm D02 had the second-highest DOR (330.00; 95% CI, 82.31–1323.06) with a sensitivity of 76.9% (95% CI: 49.7–91.8%), specificity of 99.0% (95% CI: 98.4–99.4%), PPV of 40.5% (95% CI: 23.7–59.8%) and NPV of 99.8% (95% CI: 99.4–99.9%). Other algorithms exhibited PPVs lower than 38.0%. Of 1086 algorithms other than the original 128 algorithms (algorithm set E), 4 algorithms had the same or higher PPV as well as the same or higher sensitivity and specificity than those of Algorithm D02. Of these four algorithms, one algorithm (E0624) which omitted Item 5 (presence of medical encounters for mucocutaneous lesions from SJS/TEN) achieved higher sensitivity (84.6%), PPV (42.8%) and NPV (99.9%) than Algorithm D02 (Table 4 and S8 Table). Three algorithms (E0279, E0591, and E0720) had the same performance as Algorithm D02. Of the other 1082 algorithms, 51 algorithms had higher DOR with lower sensitivity than Algorithm D02 and all the others had lower performance (S13 Table).

**Table 3:** Sensitivity, specificity, positive predictive value, negative predictive value, and diagnostic odds ratio of 37 algorithms applicable to at least one case or control patient.

Algorithm No.	TP	FN	Weighted FP	(Net FP)	Weighted TN	(Net TN)	Sensitivity	(95% CI)	Specificity	(95% CI)	PPV	(95% CI)	NPV	(95% CI)	DOR	(95% CI)
A02	7	6	14.72	(1)	1457.28	(99)	53.9	(29.1–76.8)	99.0	(98.4–99.4)	32.2	(16.6–53.2)	99.6	(99.1–99.8)	115.50	(34.61–385.49)
A09	1	12	0	(0)	1472.00	(100)	7.7	(1.4–33.3)	100.0	(99.7–100.0)	100.0	(20.7–100.0)	99.2	(98.6–99.5)	353.40	(13.72–9102.13)
A10	0	13	14.72	(1)	1457.28	(99)	0.0	(0.0–22.8)	99.0	(98.4–99.4)	0.0	(0.0–20.7)	99.1	(98.5–99.5)	3.55	(0.20–62.41)
B01	1	12	0	(0)	1472.00	(100)	7.7	(1.4–33.3)	100.0	(99.7–100.0)	100.0	(20.7–100.0)	99.2	(98.6–99.5)	353.40	(13.72–9102.13)
B02	9	4	14.72	(1)	1457.28	(99)	69.2	(42.4–87.3)	99.0	(98.4–99.4)	37.9	(21.4–57.8)	99.7	(99.3–99.9)	222.75	(61.63–805.03)
B04	1	12	14.72	(1)	1457.28	(99)	7.7	(1.4–33.3)	99.0	(98.4–99.4)	6.4	(1.1–28.7)	99.2	(98.6–99.5)	8.25	(1.01–67.61)
B06	1	12	14.72	(1)	1457.28	(99)	7.7	(1.4–33.3)	99.0	(98.4–99.4)	6.4	(1.1–28.7)	99.2	(98.6–99.5)	8.25	(1.01–67.61)
B07	0	13	14.72	(1)	1457.28	(99)	0.0	(0.0–22.8)	99.0	(98.4–99.4)	0.0	(0.0–20.7)	99.1	(98.5–99.5)	3.55	(0.20–62.41)
B08	0	13	29.44	(2)	1442.56	(98)	0.0	(0.0–22.8)	98.0	(97.2–98.6)	0.0	(0.0–11.5)	99.1	(98.5–99.5)	1.79	(0.10–30.73)
B09	1	12	0	(0)	1472.00	(100)	7.7	(1.4–33.3)	100.0	(99.7–100.0)	100.0	(20.7–100.0)	99.2	(98.6–99.5)	353.40	(13.72–9102.13)
B10	0	13	14.72	(1)	1457.28	(99)	0.0	(0.0–22.8)	99.0	(98.4–99.4)	0.0	(0.0–20.7)	99.1	(98.5–99.5)	3.55	(0.20–62.41)
B12	0	13	44.16	(3)	1427.84	(97)	0.0	(0.0–22.8)	97.0	(96.0–97.8)	0.0	(0.0–8.0)	99.1	(98.5–99.5)	1.18	(0.07–20.24)
B13	0	13	14.72	(1)	1457.28	(99)	0.0	(0.0–22.8)	99.0	(98.4–99.4)	0.0	(0.0–20.7)	99.1	(98.5–99.5)	3.55	(0.20–62.41)
B14	0	13	14.72	(1)	1457.28	(99)	0.0	(0.0–22.8)	99.0	(98.4–99.4)	0.0	(0.0–20.7)	99.1	(98.5–99.5)	3.55	(0.20–62.41)
B15	0	13	73.60	(5)	1398.40	(95)	0.0	(0.0–22.8)	95.0	(93.8–96.0)	0.0	(0.0–5.0)	99.1	(98.4–99.5)	0.70	(0.04–11.88)
B16	0	13	323.84	(22)	1148.16	(78)	0.0	(0.0–22.8)	78.0	(75.8–80.0)	0.0	(0.0–1.2)	98.9	(98.1–99.3)	0.13	(0.01–2.21)
B20	0	13	14.72	(1)	1457.28	(99)	0.0	(0.0–22.8)	99.0	(98.4–99.4)	0.0	(0.0–20.7)	99.1	(98.5–99.5)	3.55	(0.20–62.41)
B24	0	13	73.60	(5)	1398.40	(95)	0.0	(0.0–22.8)	95.0	(93.8–96.0)	0.0	(0.0–5.0)	99.1	(98.4–99.5)	0.70	(0.04–11.88)
B28	0	13	14.72	(1)	1457.28	(99)	0.0	(0.0–22.8)	99.0	(98.4–99.4)	0.0	(0.0–20.7)	99.1	(98.5–99.5)	3.55	(0.20–62.41)
B30	0	13	14.72	(1)	1457.28	(99)	0.0	(0.0–22.8)	99.0	(98.4–99.4)	0.0	(0.0–20.7)	99.1	(98.5–99.5)	3.55	(0.20–62.41)
B31	0	13	73.60	(5)	1398.40	(95)	0.0	(0.0–22.8)	95.0	(93.8–96.0)	0.0	(0.0–5.0)	99.1	(98.4–99.5)	0.70	(0.04–11.88)
B32	0	13	706.56	(48)	765.44	(52)	0.0	(0.0–22.8)	52.0	(49.5–54.5)	0.0	(0.0–0.5)	98.3	(97.2–99.0)	0.04	(0.00–0.68)
C02	7	6	14.72	(1)	1457.28	(99)	53.9	(29.1–76.8)	99.0	(98.4–99.4)	32.2	(16.6–53.2)	99.6	(99.1–99.8)	115.50	(34.61–385.49)
C10	1	12	14.72	(1)	1457.28	(99)	7.7	(1.4–33.3)	99.0	(98.4–99.4)	6.4	(1.1–28.7)	99.2	(98.6–99.5)	8.25	(1.01–67.61)
D02	10	3	14.72	(1)	1457.28	(99)	76.9	(49.7–91.8)	99.0	(98.4–99.4)	40.5	(23.7–59.8)	99.8	(99.4–99.9)	330.00	(82.31–1323.06)
D04	1	12	14.72	(1)	1457.28	(99)	7.7	(1.4–33.3)	99.0	(98.4–99.4)	6.4	(1.1–28.7)	99.2	(98.6–99.5)	8.25	(1.01–67.61)
D06	1	12	14.72	(1)	1457.28	(99)	7.7	(1.4–33.3)	99.0	(98.4–99.4)	6.4	(1.1–28.7)	99.2	(98.6–99.5)	8.25	(1.01–67.61)
D08	0	13	44.16	(3)	1427.84	(97)	0.0	(0.0–22.8)	97.0	(96.0–97.8)	0.0	(0.0–8.0)	99.1	(98.5–99.5)	1.18	(0.07–20.24)
D10	1	12	14.72	(1)	1457.28	(99)	7.7	(1.4–33.3)	99.0	(98.4–99.4)	6.4	(1.1–28.7)	99.2	(98.6–99.5)	8.25	(1.01–67.61)
D12	0	13	44.16	(3)	1427.84	(97)	0.0	(0.0–22.8)	97.0	(96.0–97.8)	0.0	(0.0–8.0)	99.1	(98.5–99.5)	1.18	(0.07–20.24)
D14	0	13	29.44	(2)	1442.56	(98)	0.0	(0.0–22.8)	98.0	(97.2–98.6)	0.0	(0.0–11.5)	99.1	(98.5–99.5)	1.79	(0.10–30.73)
D16	0	13	397.44	(27)	1074.56	(73)	0.0	(0.0–22.8)	73.0	(70.7–75.2)	0.0	(0.0–1.0)	98.8	(98.0–99.3)	0.10	(0.01–1.69)
D20	0	13	14.72	(1)	1457.28	(99)	0.0	(0.0–22.8)	99.0	(98.4–99.4)	0.0	(0.0–20.7)	99.1	(98.5–99.5)	3.55	(0.20–62.41)
D24	0	13	73.60	(5)	1398.40	(95)	0.0	(0.0–22.8)	95.0	(93.8–96.0)	0.0	(0.0–5.0)	99.1	(98.4–99.5)	0.70	(0.04–11.88)
D28	0	13	14.72	(1)	1457.28	(99)	0.0	(0.0–22.8)	99.0	(98.4–99.4)	0.0	(0.0–20.7)	99.1	(98.5–99.5)	3.55	(0.20–62.41)
D30	0	13	14.72	(1)	1457.28	(99)	0.0	(0.0–22.8)	99.0	(98.4–99.4)	0.0	(0.0–20.7)	99.1	(98.5–99.5)	3.55	(0.20–62.41)
D32	0	13	780.16	(53)	691.84	(47)	0.0	(0.0–22.8)	47.0	(44.5–49.6)	0.0	(0.0–0.5)	98.2	(96.9–98.9)	0.03	(0.00–0.55)

TP, true positive; FP, false positive; TN, true negative; FN, false negative; PPV, positive predictive value; NPV, negative predictive value; DOR, diagnostic odds ratio.

**Table 4:** Sensitivity, specificity, positive predictive value, negative predictive value, and diagnostic odds ratio of algorithm set E.

Algorithm No.	TP	FN	Weighted FP	(Net FP)	Weighted TN	(Net TN)	Sensitivity	(95% CI)	Specificity	(95% CI)	PPV	(95% CI)	NPV	(95% CI)	DOR	(95% CI)
E0279	10	3	14.72	(1)	1457.28	(99)	76.9	(49.7–91.8)	99.0	(98.4–99.4)	40.5	(23.7–59.8)	99.8	(99.4–99.9)	330.00	(82.31–1323.06)
E0591	10	3	14.72	(1)	1457.28	(99)	76.9	(49.7–91.8)	99.0	(98.4–99.4)	40.5	(23.7–59.8)	99.8	(99.4–99.9)	330.00	(82.31–1323.06)
E0624	11	2	14.72	(1)	1457.28	(99)	84.6	(57.8–95.7)	99.0	(98.4–99.4)	42.8	(25.9–61.6)	99.9	(99.5–100.0)	544.50	(110.85–2674.68)
E0720	10	3	14.72	(1)	1457.28	(99)	76.9	(49.7–91.8)	99.0	(98.4–99.4)	40.5	(23.7–59.8)	99.8	(99.4–99.9)	330.00	(82.31–1323.06)

TP, true positive; FP, false positive; TN, true negative; FN, false negative; PPV, positive predictive value; NPV, negative predictive value; DOR, diagnostic odds ratio.

Only algorithms with the same or higher performance than Algorithm D02 are shown.

## Discussion

We developed algorithms to identify SJS/TEN patients from a university hospital EMR. The algorithms consisted of the ICD-10 diagnosis codes, clinical course for SJS/TEN, medical encounters for mucocutaneous lesions from SJS/TEN, and items to exclude PNP. One algorithm, D02, achieved a moderate PPV with high sensitivity (76.9%) and high specificity (99.0%) and was considered to provide good performance in detecting SJS/TEN from EMR databases. Algorithms A09, B01, and B09 had a PPV of 100.0%, but sensitivity (7.7%) was considered too low given that the high possibility that true cases would be overlooked, increasing the rate of FN.

A systematic review of the literature regarding criteria to identify EM or SJS/TEN from 1990 to 2010 identified four articles that reported ICD-9 code-based algorithms, with PPVs of 54–60% [8]. However, these studies were conducted 17 or more years previously, and the ICD-9 code is no longer in use in Japan. A US study reported the difficulty of identifying SJS/TEN only by ICD-9-CM code within administrative claims databases, and the PPVs of the codes were only 2.5–7.0% [9]. Another US validation study using HMO data showed a PPV of 50% for hospitalized patients with ICD-9 codes of SJS/TEN or EMM, but the authors reported that the PPV was calculated by regarding SJS, TEN, and EMM as TP, and was therefore overestimated [10]. We used these previous results to develop algorithms which used not only diagnosis codes but also medication and procedure orders to more accurately identify patients with SJS/TEN. A recent study in the UK Clinical Practice Research Datalink (CPRD) developed criteria to determine SJS/TEN cases with a PPV of 87% [11]. Note that sensitivity and specificity were not calculated in that study because its population was limited to the patients sampled from CPRD based on the disease codes, and the total number of true SJS/TEN cases in the database was unknown. Additionally, it took no consideration of EMM, and its results cannot simply be compared with our data. We considered that the performance of our algorithms was better evaluated by calculating not only PPV but also sensitivity, specificity and NPV. Moreover, we also determined the PPVs for the patient population with the ICD-10 codes of diseases requiring differential diagnosis from SJS/TEN, given that several diseases (including EMM) require differentiation from SJS/TEN when identifying true cases in the EMR database.

Although Algorithm D02 appeared to have sufficient performance, with high sensitivity, specificity, and NPV, its moderate PPV (40.5%) was probably due to the very low incidence of SJS/TEN in this population (<1.0%). PPV is strongly affected by the prevalence of the target disease, in contrast to sensitivity and specificity [32]. Algorithm D02 identified only one FP case, which was in fact a case of EMM. EMM is often misdiagnosed as SJS/TEN but represents a different clinical entity. Because EMM was also rare, increasing the number of true cases might have improved the validity of performance indices, such as an increased PPV, via a relative decrease in FP. However, this would require a multi-institutional study, and the increased cost is unlikely to match the expected performance improvement. Pathological findings are necessary to differentiate SJS/TEN and EMM patients, but claims databases do not include such information.

Algorithm D02 determined 3 SJS/TEN cases as FN, of which 1 TEN case did not undergo skin biopsy (Item 3), 1 SJS case did not receive systemic treatment for SJS/TEN (Item 4), and 1 TEN patient had no record of medical encounters for mucocutaneous lesions from SJS/TEN (Item 5) (S11 Table). The Japanese clinical guidelines for SJS/TEN require a histopathological finding of epidermal necrosis to establish a diagnosis of SJS [2], and skin biopsy is considered mandatory in our algorithm. To diagnose TEN, however, the guidelines do not require skin biopsy, but merely consider it helpful. A prior cross-sectional study of 287 SJS/TEN cases in Japan showed that 2.8% did not receive systemic treatment for SJS/TEN [2], and Algorithm D02 would not identify such cases. A nationwide study in Japan showed that 23% of TEN cases did not have mucous membrane lesions, and hence Algorithm D02 would not be able to detect these cases either [3].

It is possible that Algorithm D02 may mistakenly identify the following six diseases as SJS/TEN: bullous pemphigoid (BP), Behçet’s disease, Kawasaki disease, oral lichen planus, systemic lupus erythematosus (SLE), and SJS/TEN-like reactions to anti-PD-1 therapy [33]. BP is the most common form among autoimmune blistering diseases and primarily affects the elderly [34]. Enzyme-linked immunosorbent assay (ELISA) to detect anti-BP180 antibody is examined at multiple times during the treatment [35]. Algorithm D02 requires multiple testing (≥2) to exclude BP and its diagnostic performance may be superior to that of the original algorithm. Cases of these rare diseases would almost certainly not be diagnosed as SJS/TEN, and their inclusion as diseases requiring differential diagnosis appears unnecessary.

Of algorithm set E, developed by reducing items from the full 6-item algorithm sets A–D, E0624 had higher performance than Algorithm D02, indicating that omitting Item 5 (presence of medical encounters for mucocutaneous lesions from SJS/TEN) may result in better performance. In fact, one patient with atypical TEN case presenting no mucocutaneous lesions was positive with Algorithm E0624. On the other hand, the algorithms lacking Item 5 such as E0624 may fail to exclude cases with impetigo, DIHS, and SSSS and inadvertently result in an increase in FPs when applied in different settings because impetigo, DIHS, and SSSS generally have no mucocutaneous involvement. Six patients with impetigo and one with DIHS in the present study were incidentally ruled out by Item 3 (presence of skin biopsy). Thus, we recommend Algorithm D02 for future application, however, a further testing in a wider population is warranted. Algorithm D02 had the same diagnostic performance as Algorithms E0279, 0591, and 0720, which commonly include Items 3–5, indicating that Item 2 (hospitalization) and Item 6b (number of anti-Dsg1 or Dsg3 antibody tests) did not influence diagnostic performance in this study. However, routine care for SJS/TEN requires hospitalization, and Item 6b is accordingly required to exclude patients with PNP. These two items are therefore considered necessary when the algorithm is applied to other populations.

The degree of sensitivity, specificity, and PPV an algorithm requires depends on the purpose for which it was developed. Because an algorithm with high specificity is rarely positive in the absence of disease, a highly specific algorithm is useful in confirming a diagnosis that has been suggested by other data [36]. On this basis, Algorithm D02 appears useful in establishing the differential diagnosis between SJS/TEN and other diseases which require differentiation from SJS/TEN. Further, with high specificity, the estimates of risk ratio will not be biased when the sensitivity of disease misclassification is nondifferential [37]. The almost perfect specificity of Algorithm D02 will therefore prevent bias in database studies aiming to estimate relative risks of SJS/TEN.

We recommend that Algorithm D02 be utilized in epidemiologic studies of SJS/TEN using claims databases as follows. First, patients with the diagnosis codes shown in S1 Table, recorded at the Department of Dermatology, should be subject to the algorithm. Second, our limited experience may suggest the following time intervals of algorithm items for practical application: patients with SJS/TEN should meet Items 2–4 within one week after the date of ICD-10 diagnosis shown in S1 Table; Item 5 should be met within two weeks before or after the diagnosis date; and Item 6b should not be met. These suggested time intervals should be further examined in practice settings.

Three limitations of this study warrant mention. First, the study used data from a single university hospital and does not necessarily represent patients with SJS/TEN in Japan. Other medical facilities may employ different procedures and treatment options. Nevertheless, all items used in the algorithms including D02, other than slit-lamp microscopy, were consistent with the clinical practice guidelines for SJS/TEN and therefore seem to represent the typical clinical course of SJS/TEN in Japan [2]. Given that SJS/TEN cases in Japan are correctly diagnosed and treated at dermatology divisions staffed with experts, e.g. in teaching hospitals, we believe that Algorithm D02 may be most applicable to true SJS/TEN cases found at other tertiary medical care hospitals, where most SJS/TEN cases are transferred and treated. In addition, these items are coded by the standard reimbursement codes under the universal national healthcare insurance coverage and are commonly available in many kinds of healthcare database in Japan [38]. Thus, a degree of generalizability of the algorithm may be expected in the similar clinical setting to the ones where it was developed [39, 40]. Reproducibility of the present results may be limited in the other settings including secondary-care hospitals and claims-based databases, considering the small sample size in the present study. Second, patients with ICD-10 codes for TSS, SSSS, AGEP, PNP, or nonbullous erythema multiforme were not sampled as control patients, because of the scarcity of these patients. When Algorithm D02 was applied to patients with these diseases in the pilot study, it ruled them out (unpublished data). Third, we did not consider medical records of outpatient visits or hospitalizations for patients transferred from other hospitals.

## Conclusions

We developed EMR-based algorithms and showed that one (Algorithm D02) had moderate PPV and high specificity for SJS/TEN cases based on the EMRs of a tertiary university hospital. This algorithm warrants further testing in different settings.

## Supporting information

S1 Table ICD-10 codes to identify control patients with diseases other than Stevens-Johnson syndrome or toxic epidermal necrolysis. ICD-10, International Classification of Diseases, 10th Edition.
(DOCX)

S2 Table ICD-10 codes of mucocutaneous lesions from Stevens-Johnson syndrome and toxic epidermal necrolysis. ICD-10, International Classification of Diseases, 10th Edition.
(DOCX)

S3 Table Pattern of algorithm set A. (DOCX)

S4 Table Pattern of algorithm set B. (DOCX)

S5 Table Pattern of algorithm set C. (DOCX)

S6 Table Pattern of algorithm set D. (DOCX)

S7 Table Number of algorithms in algorithm set E. Eq. (1), where seven is the number of items (Item 1–6b). Eq. (5), where 64 is the number of algorithms in algorithm sets B and D. Eq. (6), where 64 is the number of algorithms in algorithm sets A and C. Eq. (1) to (6), where 2^n^ is the number of combinations of n dichotomous items. Eq. (2) to (5), where C(6, n) is the number of combinations for n items out of six (Item 1–6), and C(5, n) is the number of combinations for n items out of five (Item 1–5). Eq. (2) to (6), where the number two is the pattern of Item 6 (6a or 6b).
(DOCX)

S8 Table Pattern of algorithm set E. (XLSX)

S9 Table Distributions of ICD-10 codes in case patients. ICD-10, International Classification of Diseases, 10th Edition. Data are presented as the number of cases (multiple diagnosis included). ^a^ ICD-10 code diagnosis only for the purpose of testing.
(DOCX)

S10 Table Distribution of ICD-10 codes in control candidates and sampled control patients. ICD-10, International Classification of Diseases, 10th Edition. Data are presented as the number of control candidates and controls (multiple diagnoses included).
(DOCX)

S11 Table Chronological order of algorithm items met in true case patients. ICD-10, International Classification of Diseases, 10th Edition; SJS, Stevens-Johnson syndrome; TEN, toxic epidermal necrolysis; DIHS, drug-induced hypersensitivity syndrome. Data on orders are presented as the order of algorithm items which were met, and data on days are presented as the number of days from the recorded date on which the first algorithm item was met. ^a^ First recorded diagnosis. ^b^ ICD-10 code diagnosis only for the purpose of testing.
(DOCX)

S12 Table Performance of 91 algorithms applicable to neither case nor control patients. TP, true positive; FP, false positive; TN, true negative; FN, false negative; PPV, positive predictive value; NPV, negative predictive value; DOR, diagnostic odds ratio.
(XLSX)

S13 Table Performance of algorithm set E. TP, true positive; FP, false positive; TN, true negative; FN, false negative; PPV, positive predictive value; NPV, negative predictive value; DOR, diagnostic odds ratio.
(XLSX)
