# Supplementary material for: Development of an electronic medical record-based algorithm to identify patients with Stevens-Johnson syndrome and toxic epidermal necrolysis in Japan
Source: PLoS One. 2019 Aug 13;14(8):e0221130. doi: 10.1371/journal.pone.0221130 (PMC6692049; doi:10.1371/journal.pone.0221130)
Supplement: S1 Table — ICD-10, International Classification of Diseases, 10th Edition. (DOCX) [file pone.0221130.s001.docx]

**S1 Table.** **ICD-10 codes to identify control patients with diseases other than Stevens-Johnson syndrome or toxic epidermal necrolysis.**

| ICD-10 code | Diagnosis name |
| --- | --- |
| A48.3 | Toxic shock syndrome |
| L00 | Staphylococcal scalded skin syndrome |
| L01 | Impetigo, excluding eosinophilic pustular folliculitis |
| L08.0 | Acute generalized exanthematous pustulosis |
| L10.8 | Paraneoplastic pemphigus |
| L27.0 | Generalized skin eruption due to drugs and medicaments, excluding lupus erythematosus and steroid-induced dermatitis |
| L27.1 | Localized skin eruption due to drugs and medicaments |
| L27.9 | Toxicoderma |
| L51.0 | Nonbullous erythema multiforme |
| L51.1 | Bullous erythema multiforme and Stevens-Johnson syndrome |
| L51.2 | Toxic epidermal necrolysis [Lyell] |
| L51.8 | Other erythema multiforme |
| L51.9 | Erythema multiforme, unspecified |

ICD-10, International Classification of Diseases, 10th Edition.
