# Supplementary material for: Development of an electronic medical record-based algorithm to identify patients with Stevens-Johnson syndrome and toxic epidermal necrolysis in Japan
Source: PLoS One. 2019 Aug 13;14(8):e0221130. doi: 10.1371/journal.pone.0221130 (PMC6692049; doi:10.1371/journal.pone.0221130)
Supplement: S2 Table — ICD-10, International Classification of Diseases, 10th Edition. (DOCX) [file pone.0221130.s002.docx]

**S2 Table.** **ICD-10 codes of mucocutaneous lesions from Stevens-Johnson syndrome and toxic epidermal necrolysis.**

| ICD-10 code | Diagnosis name |
| --- | --- |
| H10.2 | Pseudomembranous conjunctivitis and Medicamentosa conjunctivitis |
| H10.3 | Acute conjunctivitis |
| H10.8 | Conjunctival erosion and Conjunctival ulcer |
| H10.9 | Conjunctivitis |
| H11.2 | Symblepharon |
| H11.4 | Conjunctival injection |
| H16.0 | Corneal erosion |
| H16.1 | Corneal epithelial erosion, Diffuse punctate keratitis, Superficial punctate keratitis, Superficial keratitis, and Superficial punctate keratopathy |
| H16.2 | Keratoconjunctival erosion, Keratoconjunctivitis, Acute keratoconjunctivitis, and Medicamentosa keratoconjunctivitis |
| H16.8 | Medicamentosa keratitis |
| H16.9 | Keratitis and Acute keratitis |
| H18.8 | Corneal epithelial abrasion, Corneal abrasion, Multiple corneal erosion, and Corneal epithelial defect |
| H18.9 | Injectio pericornealis |
| K12.1 | Stomatitis, Hemorrhagic stomatitis, Vesicular stomatitis, Multiple stomatitis, Ulcerative stomatitis, and Intractable stomatitis |
| K12.3 | Oral mucositis (ulcerative) |
| K13.0 | Perleche, Angular stomatitis, Angular cheilitis, Lip erosion, Cheilitis, and Canker sore |
| K14.0 | Tongue erosion, Glossitis, and Tongue ulcer |
| K62.8 | Anal erosion |
| N48.5 | Ulcer of penis |
| N76.2 | Vulvitis |
| N86 | Cervicovaginal erosion and Cervicovaginal pseudoerosion |
| S05.0 | Corneal epithelial wound and Superficial conjunctivitis wound |

ICD-10, International Classification of Diseases, 10th Edition.
