# Supplementary material for: Development of an electronic medical record-based algorithm to identify patients with Stevens-Johnson syndrome and toxic epidermal necrolysis in Japan
Source: PLoS One. 2019 Aug 13;14(8):e0221130. doi: 10.1371/journal.pone.0221130 (PMC6692049; doi:10.1371/journal.pone.0221130)
Supplement: S3 Table — (DOCX) [file pone.0221130.s003.docx]

**S3 Table.** **Pattern of algorithm set A.**

| Algorithm No. | Item 1 | Item 2 | Item 3 | Item 4 | Item 5 | Item 6a |
| --- | --- | --- | --- | --- | --- | --- |
| A01 | yes | yes | yes | yes | yes | yes |
| A02 | yes | yes | yes | yes | yes | no |
| A03 | yes | yes | yes | yes | no | yes |
| A04 | yes | yes | yes | yes | no | no |
| A05 | yes | yes | yes | no | yes | yes |
| A06 | yes | yes | yes | no | yes | no |
| A07 | yes | yes | yes | no | no | yes |
| A08 | yes | yes | yes | no | no | no |
| A09 | yes | yes | no | yes | yes | yes |
| A10 | yes | yes | no | yes | yes | no |
| A11 | yes | yes | no | yes | no | yes |
| A12 | yes | yes | no | yes | no | no |
| A13 | yes | yes | no | no | yes | yes |
| A14 | yes | yes | no | no | yes | no |
| A15 | yes | yes | no | no | no | yes |
| A16 | yes | yes | no | no | no | no |
| A17 | yes | no | yes | yes | yes | yes |
| A18 | yes | no | yes | yes | yes | no |
| A19 | yes | no | yes | yes | no | yes |
| A20 | yes | no | yes | yes | no | no |
| A21 | yes | no | yes | no | yes | yes |
| A22 | yes | no | yes | no | yes | no |
| A23 | yes | no | yes | no | no | yes |
| A24 | yes | no | yes | no | no | no |
| A25 | yes | no | no | yes | yes | yes |
| A26 | yes | no | no | yes | yes | no |
| A27 | yes | no | no | yes | no | yes |
| A28 | yes | no | no | yes | no | no |
| A29 | yes | no | no | no | yes | yes |
| A30 | yes | no | no | no | yes | no |
| A31 | yes | no | no | no | no | yes |
| A32 | yes | no | no | no | no | no |
