# Supplementary material for: Development of an electronic medical record-based algorithm to identify patients with Stevens-Johnson syndrome and toxic epidermal necrolysis in Japan
Source: PLoS One. 2019 Aug 13;14(8):e0221130. doi: 10.1371/journal.pone.0221130 (PMC6692049; doi:10.1371/journal.pone.0221130)
Supplement: S4 Table — (DOCX) [file pone.0221130.s004.docx]

**S4 Table. Pattern of algorithm set B.**

| Algorithm No. | Item 2 | Item 3 | Item 4 | Item 5 | Item 6a |
| --- | --- | --- | --- | --- | --- |
| B01 | yes | yes | yes | yes | yes |
| B02 | yes | yes | yes | yes | no |
| B03 | yes | yes | yes | no | yes |
| B04 | yes | yes | yes | no | no |
| B05 | yes | yes | no | yes | yes |
| B06 | yes | yes | no | yes | no |
| B07 | yes | yes | no | no | yes |
| B08 | yes | yes | no | no | no |
| B09 | yes | no | yes | yes | yes |
| B10 | yes | no | yes | yes | no |
| B11 | yes | no | yes | no | yes |
| B12 | yes | no | yes | no | no |
| B13 | yes | no | no | yes | yes |
| B14 | yes | no | no | yes | no |
| B15 | yes | no | no | no | yes |
| B16 | yes | no | no | no | no |
| B17 | no | yes | yes | yes | yes |
| B18 | no | yes | yes | yes | no |
| B19 | no | yes | yes | no | yes |
| B20 | no | yes | yes | no | no |
| B21 | no | yes | no | yes | yes |
| B22 | no | yes | no | yes | no |
| B23 | no | yes | no | no | yes |
| B24 | no | yes | no | no | no |
| B25 | no | no | yes | yes | yes |
| B26 | no | no | yes | yes | no |
| B27 | no | no | yes | no | yes |
| B28 | no | no | yes | no | no |
| B29 | no | no | no | yes | yes |
| B30 | no | no | no | yes | no |
| B31 | no | no | no | no | yes |
| B32 | no | no | no | no | no |
