# Supplementary material for: Development of an electronic medical record-based algorithm to identify patients with Stevens-Johnson syndrome and toxic epidermal necrolysis in Japan
Source: PLoS One. 2019 Aug 13;14(8):e0221130. doi: 10.1371/journal.pone.0221130 (PMC6692049; doi:10.1371/journal.pone.0221130)
Supplement: S5 Table — (DOCX) [file pone.0221130.s005.docx]

**S5 Table. Pattern of algorithm set C.**

| Algorithm No. | Item 1 | Item 2 | Item 3 | Item 4 | Item 5 | Item 6b |
| --- | --- | --- | --- | --- | --- | --- |
| C01 | yes | yes | yes | yes | yes | yes |
| C02 | yes | yes | yes | yes | yes | no |
| C03 | yes | yes | yes | yes | no | yes |
| C04 | yes | yes | yes | yes | no | no |
| C05 | yes | yes | yes | no | yes | yes |
| C06 | yes | yes | yes | no | yes | no |
| C07 | yes | yes | yes | no | no | yes |
| C08 | yes | yes | yes | no | no | no |
| C09 | yes | yes | no | yes | yes | yes |
| C10 | yes | yes | no | yes | yes | no |
| C11 | yes | yes | no | yes | no | yes |
| C12 | yes | yes | no | yes | no | no |
| C13 | yes | yes | no | no | yes | yes |
| C14 | yes | yes | no | no | yes | no |
| C15 | yes | yes | no | no | no | yes |
| C16 | yes | yes | no | no | no | no |
| C17 | yes | no | yes | yes | yes | yes |
| C18 | yes | no | yes | yes | yes | no |
| C19 | yes | no | yes | yes | no | yes |
| C20 | yes | no | yes | yes | no | no |
| C21 | yes | no | yes | no | yes | yes |
| C22 | yes | no | yes | no | yes | no |
| C23 | yes | no | yes | no | no | yes |
| C24 | yes | no | yes | no | no | no |
| C25 | yes | no | no | yes | yes | yes |
| C26 | yes | no | no | yes | yes | no |
| C27 | yes | no | no | yes | no | yes |
| C28 | yes | no | no | yes | no | no |
| C29 | yes | no | no | no | yes | yes |
| C30 | yes | no | no | no | yes | no |
| C31 | yes | no | no | no | no | yes |
| C32 | yes | no | no | no | no | no |
