# Supplementary material for: Development of an electronic medical record-based algorithm to identify patients with Stevens-Johnson syndrome and toxic epidermal necrolysis in Japan
Source: PLoS One. 2019 Aug 13;14(8):e0221130. doi: 10.1371/journal.pone.0221130 (PMC6692049; doi:10.1371/journal.pone.0221130)
Supplement: S6 Table — (DOCX) [file pone.0221130.s006.docx]

**S6 Table. Pattern of algorithm set D.**

| Algorithm No. | Item 2 | Item 3 | Item 4 | Item 5 | Item 6b |
| --- | --- | --- | --- | --- | --- |
| D01 | yes | yes | yes | yes | yes |
| D02 | yes | yes | yes | yes | no |
| D03 | yes | yes | yes | no | yes |
| D04 | yes | yes | yes | no | no |
| D05 | yes | yes | no | yes | yes |
| D06 | yes | yes | no | yes | no |
| D07 | yes | yes | no | no | yes |
| D08 | yes | yes | no | no | no |
| D09 | yes | no | yes | yes | yes |
| D10 | yes | no | yes | yes | no |
| D11 | yes | no | yes | no | yes |
| D12 | yes | no | yes | no | no |
| D13 | yes | no | no | yes | yes |
| D14 | yes | no | no | yes | no |
| D15 | yes | no | no | no | yes |
| D16 | yes | no | no | no | no |
| D17 | no | yes | yes | yes | yes |
| D18 | no | yes | yes | yes | no |
| D19 | no | yes | yes | no | yes |
| D20 | no | yes | yes | no | no |
| D21 | no | yes | no | yes | yes |
| D22 | no | yes | no | yes | no |
| D23 | no | yes | no | no | yes |
| D24 | no | yes | no | no | no |
| D25 | no | no | yes | yes | yes |
| D26 | no | no | yes | yes | no |
| D27 | no | no | yes | no | yes |
| D28 | no | no | yes | no | no |
| D29 | no | no | no | yes | yes |
| D30 | no | no | no | yes | no |
| D31 | no | no | no | no | yes |
| D32 | no | no | no | no | no |
