# Supplementary material for: Development of an electronic medical record-based algorithm to identify patients with Stevens-Johnson syndrome and toxic epidermal necrolysis in Japan
Source: PLoS One. 2019 Aug 13;14(8):e0221130. doi: 10.1371/journal.pone.0221130 (PMC6692049; doi:10.1371/journal.pone.0221130)
Supplement: S7 Table — Eq. (1), where seven is the number of items (Item 1–6b). Eq. (5), where 64 is the number of algorithms in algorithm sets B and D. Eq. (6), where 64 is the number of algorithms in algorithm sets A and C. Eq. (1) to (6), where 2n is the number of combinations of n dichotomous items. Eq. (2) to (5), where C(6, n) is the number of combinations for n items out of six (Item 1–6), and C(5, n) is the number of combinations for n items out of five (Item 1–5). Eq. (2) to (6), where the number two is the pattern of Item 6 (6a or 6b). (DOCX) [file pone.0221130.s007.docx]

**S7 Table. Number of algorithms in algorithm set E.**

| Number of items used in algorithms | Number of algorithms | |
| --- | --- | --- |
| 1 | $\text{7 × }\text{2}^{\text{1}}\text{ = 14}$ | Eq. (1) |
| 2 | $\left\{ \text{C(6, 2) × 2 - C(5, 2)} \right\}\text{ × }\text{2}^{\text{2}}\text{ = 80}$ | Eq. (2) |
| 3 | $\left\{ \text{C(6, 3) × 2 - C(5, 3)} \right\}\text{ × }\text{2}^{\text{3}}\text{ = 240}$ | Eq. (3) |
| 4 | $\left\{ \text{C(6, 4) × 2 - C(5, 4)} \right\}\text{ × }\text{2}^{\text{4}}\text{ = 400}$ | Eq. (4) |
| 5 | $\left\{ \text{C(6, 5) × 2 - C(5, 5)} \right\}\text{ × }\text{2}^{\text{5}}\text{ - 64 = 288}$ | Eq. (5) |
| 6 | $\text{2 × 2}^{\text{6}}\text{ - 64 = 64}$ | Eq. (6) |
| Total | 1086 |  |

Eq. (1), where seven is the number of items (Item 1–6b).

Eq. (5), where 64 is the number of algorithms in algorithm sets B and D.

Eq. (6), where 64 is the number of algorithms in algorithm sets A and C.

Eq. (1) to (6), where 2^n^ is the number of combinations of n dichotomous items.

Eq. (2) to (5), where C(6, n) is the number of combinations for n items out of six (Item 1–6), and C(5, n) is the number of combinations for n items out of five (Item 1–5).

Eq. (2) to (6), where the number two is the pattern of Item 6 (6a or 6b).
