# Supplementary material for: Development of an electronic medical record-based algorithm to identify patients with Stevens-Johnson syndrome and toxic epidermal necrolysis in Japan
Source: PLoS One. 2019 Aug 13;14(8):e0221130. doi: 10.1371/journal.pone.0221130 (PMC6692049; doi:10.1371/journal.pone.0221130)
Supplement: S9 Table — ICD-10, International Classification of Diseases, 10th Edition. Data are presented as the number of cases (multiple diagnosis included). a ICD-10 code diagnosis only for the purpose of testing. (DOCX) [file pone.0221130.s009.docx]

**S9 Table. Distributions of ICD-10 codes in case patients.**

| ICD-10 code | Diagnosis name | Case patient n = 13 | |
| --- | --- | --- | --- |
|  |  | n | (%) |
| A31.9 | Nontuberculous mycobacteriosis (suspected^a^) | 1 | (7.7) |
| L27.0 | Generalized skin eruption due to drugs and medicaments, excluding lupus erythematosus and steroid-induced dermatitis | 3 | (23.1) |
| L27.9 | Toxicoderma | 2 | (15.4) |
| L51.1 | Stevens-Johnson syndrome | 6 | (46.2) |
| L51.1 | Stevens-Johnson syndrome (suspected^a^) | 1 | (7.7) |
| L51.2 | Toxic epidermal necrolysis [Lyell] | 2 | (15.4) |
| L70.0 | Acne vulgaris (suspected^a^) | 1 | (7.7) |

ICD-10, International Classification of Diseases, 10th Edition.

Data are presented as the number of cases (multiple diagnosis included).

^a^ ICD-10 code diagnosis only for the purpose of testing.
