# Supplementary material for: Development of an electronic medical record-based algorithm to identify patients with Stevens-Johnson syndrome and toxic epidermal necrolysis in Japan
Source: PLoS One. 2019 Aug 13;14(8):e0221130. doi: 10.1371/journal.pone.0221130 (PMC6692049; doi:10.1371/journal.pone.0221130)
Supplement: S10 Table — ICD-10, International Classification of Diseases, 10th Edition. Data are presented as the number of control candidates and controls (multiple diagnoses included). (DOCX) [file pone.0221130.s010.docx]

**S10 Table. Distribution of ICD-10 codes in control candidates and sampled control patients.**

| ICD-10 code | Diagnosis name | Control candidate patient n = 1472 | | Control patient n = 100 | |
| --- | --- | --- | --- | --- | --- |
|  |  | n | (%) | n | (%) |
| A48.3 | Toxic shock syndrome | 0 | (0.0) | 0 | (0.0) |
| L00 | Staphylococcal scalded skin syndrome | 1 | (0.1) | 0 | (0.0) |
| L01 | Impetigo, excluding eosinophilic pustular folliculitis | 119 | (8.1) | 6 | (6.0) |
| L08.0 | Acute generalized exanthematous pustulosis | 4 | (0.3) | 0 | (0.0) |
| L10.8 | Paraneoplastic pemphigus | 5 | (0.3) | 0 | (0.0) |
| L27.0 | Generalized skin eruption due to drugs and medicaments,  excluding lupus erythematosus and steroid-induced dermatitis | 614 | (41.7) | 39 | (39.0) |
| L27.1 | Localized skin eruption due to drugs and medicaments | 31 | (2.1) | 2 | (2.0) |
| L27.9 | Toxicoderma | 687 | (46.7) | 46 | (46.0) |
| L51.0 | Nonbullous erythema multiforme | 1 | (0.1) | 0 | (0.0) |
| L51.1 | Bullous erythema multiforme and Stevens-Johnson syndrome | 11 | (0.7) | 2 | (2.0) |
| L51.2 | Toxic epidermal necrolysis [Lyell] | 0 | (0.0) | 0 | (0.0) |
| L51.8 | Other erythema multiforme | 61 | (4.1) | 6 | (6.0) |
| L51.9 | Erythema multiforme, unspecified | 65 | (4.4) | 4 | (4.0) |

ICD-10, International Classification of Diseases, 10th Edition.

Data are presented as the number of control candidates and controls (multiple diagnoses included).
