# Supplementary material for: Development of an electronic medical record-based algorithm to identify patients with Stevens-Johnson syndrome and toxic epidermal necrolysis in Japan
Source: PLoS One. 2019 Aug 13;14(8):e0221130. doi: 10.1371/journal.pone.0221130 (PMC6692049; doi:10.1371/journal.pone.0221130)
Supplement: S11 Table — ICD-10, International Classification of Diseases, 10th Edition; SJS, Stevens-Johnson syndrome; TEN, toxic epidermal necrolysis; DIHS, drug-induced hypersensitivity syndrome. Data on orders are presented as the order of algorithm items which were met, and data on days are presented as the number of days from the recorded date on which the first algorithm item was met. a First recorded diagnosis. b ICD-10 code diagnosis only for the purpose of testing. (DOCX) [file pone.0221130.s011.docx]

**S11 Table. Chronological order of algorithm items met in true case patients.**

| ID | Case | ICD-10 code diagnosis^a^ | Item 1 | | Item 2 | | Item 3 | | Item 4 | | Items 5 | | Item 6a | | Item 6b | |
| --- | --- | --- | --- | --- | --- | --- | --- | --- | --- | --- | --- | --- | --- | --- | --- | --- |
|  |  |  | order | (day) | order | (day) | order | (day) | order | (day) | order | (day) | order | (day) | order | (day) |
| 1 | SJS | SJS | 1 | (0) | 1 | (0) | 1 | (0) | 1 | (0) | 1 | (0) | N/A | (N/A) | N/A | (N/A) |
| 2 | SJS | Toxicoderma | N/A | (N/A) | 1 | (0) | 2 | (1) | 3 | (2) | 3 | (2) | N/A | (N/A) | N/A | (N/A) |
| 3 | SJS | SJS, DIHS | 1 | (0) | 1 | (0) | 1 | (0) | 1 | (0) | 2 | (3) | N/A | (N/A) | N/A | (N/A) |
| 4 | SJS | Generalized skin eruption due to drugs and medicaments | N/A | (N/A) | 2 | (1) | 2 | (1) | 2 | (1) | 1 | (0) | N/A | (N/A) | N/A | (N/A) |
| 5 | SJS | SJS | 1 | (0) | 1 | (0) | 1 | (0) | 2 | (1) | 3 | (3) | N/A | (N/A) | N/A | (N/A) |
| 6 | SJS | SJS | 1 | (0) | 1 | (0) | 1 | (0) | 1 | (0) | 1 | (0) | N/A | (N/A) | N/A | (N/A) |
| 7 | SJS | Nontuberculous mycobacteriosis (suspected^b^) | N/A | (N/A) | 1 | (0) | 2 | (15) | N/A | (N/A) | 2 | (15) | N/A | (N/A) | N/A | (N/A) |
| 8 | SJS | SJS (suspected^b^) | 2 | (11) | 1 | (0) | 2 | (11) | 3 | (12) | 2 | (11) | N/A | (N/A) | N/A | (N/A) |
| 9 | SJS | SJS | 1 | (0) | 1 | (0) | 1 | (0) | 1 | (0) | 1 | (0) | N/A | (N/A) | N/A | (N/A) |
| 10 | TEN | TEN | 3 | (26) | 2 | (2) | N/A | (N/A) | 4 | (27) | 4 | (27) | 1 | (0) | N/A | (N/A) |
| 11 | TEN | SJS | 1 | (0) | 1 | (0) | 2 | (1) | 2 | (1) | 1 | (0) | N/A | (N/A) | N/A | (N/A) |
| 12 | TEN | Acne vulgaris (suspected^b^) | N/A | (N/A) | 1 | (0) | 3 | (34) | 3 | (34) | 3 | (34) | 2 | (13) | N/A | (N/A) |
| 13 | TEN | Toxicoderma | 1 | (0) | 2 | (1) | 2 | (1) | 2 | (1) | N/A | (N/A) | N/A | (N/A) | N/A | (N/A) |

ICD-10, International Classification of Diseases, 10th Edition; SJS, Stevens-Johnson syndrome; TEN, toxic epidermal necrolysis; DIHS, drug-induced hypersensitivity syndrome.

Data on orders are presented as the order of algorithm items which were met, and data on days are presented as the number of days from the recorded date on which the first algorithm item was met.

^a^ First recorded diagnosis.

^b^ ICD-10 code diagnosis only for the purpose of testing.
